# Advice as to a Headache

**Published:** 1888-08

**Authors:** 


### ADVICE AS TO A HEADACHE.

A teacher in Tennessee, who has long suffered from headache, and has tried physicians and remedies with only temporary relief, asks our advice—as to diet and manner of living. He has a good appetite and is otherwise apparently well.

We may premise that a headache may be due to one of many causes, or to several causes acting together. A remedy suited to one case may be harmful in another. Nor can any case be cured without the removal of the cause.

Without some knowledge of the habits, the temperament, the physical tendencies and the general surroundings of a sufferer, it is impossible to mark out any scheme of diet adapted to a particular case.

But any one who is an intelligent observer of himself, and is possessed of a strong will, can, probably, treat himself as successfully as any ordinary physician can treat him.

We will address our correspondent directly, but the advice, with the necessary modifications, will do for others :

1. You are aware that vigorous out-door exercise is essential to sound health in all persons, and especially so in the case of a brain worker, under the peculiar strain of a schoolroom. You need, at the minimum, two hours a day of such exercise. Perhaps with your Southern habits and conveniences, horseback riding might profitably interchange with rapid, cheery walking.

2. You are equally aware that, at least, eight hours of solid sleep are still more essential—sleep in a well-ventilated, sun-disinfected room.

3. You may not be aware, but it is true, that "biliousness" and indigestion are generally due far more to over-eating or under-eating than to the kinds of food eaten. See if you can trace a connection between the quantity of your food and your distressing symptoms. Lessen it below the average of the past, and note whether the tendency to headache lessens with it. If it does, you are on the right track. If not, try the effect of more frequent and abundant meals.

4. Constipation fills the blood with poisons that affect the brain. Let this be remedied, if possible, by your food. Abjure white bread, and use bread made from "entire wheat flour," or mush from oatmeal or "wheat germ-meal," with a free addition of fruit.

5. Notice whether any particular article of food positively disagrees with you—in itself, in the mode of cooking it, or in the quantity eaten—and govern yourself accordingly.

Make trial of these suggestions for three months and note the result.
—*Youths' Companion.*

